# Territorial battles between fiddler crab species

**DOI:** 10.1098/rsos.160621

**Published:** 2017-01-18

**Authors:** H. L. Clark, P. R. Y. Backwell

**Affiliations:** Research School of Biology, The Australian National University, Canberra, Australian Capital Territory 0200, Australia

**Keywords:** interspecific, competition, *Uca*, fiddler crab, territory

## Abstract

Many species worldwide are impacted by habitat loss. This may result in increased competition both within species and between species. Many studies have demonstrated that when two previously non-overlapping species are forced to compete over a resource, one species is likely to become dominant over the other. This study explores the impact a larger species of fiddler crab (*Tabuca elegans*—previously known as *Uca elegans*) has when invading an area previously used solely by a smaller species (*Austruca mjoebergi*—previously known as *Uca mjoebergi*). Here we show that, while there are some detrimental effects of living next to a heterospecific, they are relatively minor. New heterospecific neighbours fight more regularly with resident crabs, but each fight is no longer or more escalated than those between the resident and a new conspecific male. The residents are not specifically targeted by intruding heterospecifics, thus, given the large advantage of having a heterospecific neighbour in terms of lowered competition for females, the overall impact of species mixing is probably not as negative as might have been predicted.

## 1. Introduction

Habitat loss is a factor limiting the distribution of many species worldwide [1,2], and has often been attributed to anthropogenic causes, such as climate change [3] and/or development [1]. Reductions in available habitat may place an upper limit on the total numbers of individuals reliant on a given space [4]. Studies on shore birds demonstrated a decline in numbers after events resulting in local habitat loss, as a result of increased intraspecific and interspecific competition [5,6]. Increased competition is costly as it results in reduced fitness or even survivorship for individuals [7] or even entire species [8].

Studies on interspecific competition have revealed that one species usually emerges as the dominant at the cost of the subordinate species [8–10]. Consequences resulting from competition include loss of valuable resources (e.g. food or shelter, [7,9]), increased exposure to predators or environmental conditions [11], or if the competitive encounters are of an aggressive nature, injury or death [12–14]. Understanding competitive processes may aid in predicting biodiversity losses associated with habitat losses and/or restrictions. Interspecific competition occurs when two species occupy the same ecological niche and require the same resource [8,9]. It is most easily studied when competitive behaviours are easily decipherable and observable [15]. Fiddler crabs are an excellent model for studying interspecific competition as space is usually limited but is extremely valuable to individuals [12,16].

Fiddler crabs live in mixed sex, high-density populations, where each individual occupies a territory containing an area of sediment surface for feeding and courtship display as well as a central burrow that is used for avoidance of predators and extreme weather, access to water, mating and incubation [17]. As a result, territories are essential for reproduction and survival of fiddler crabs [16]. Males have one greatly enlarged claw that they use to attract mates and in territorial disputes with other males [12,18]. Male crabs can either give their burrow to a female after reproduction, or may lose it through an aggressive interaction with a ‘floating’ male (a territory-less individual who wanders through the population looking for a new territory). Males that lose their territories must then either dig a new burrow (usually on the outer edges of the population since that is often the only place where undisputed space can be found), find an empty burrow or fight a resident male for a burrow [19].

Multiple fiddler crab species often use the same mudflat, and it is common for species to occur in specific intertidal zones due to preferences for sediment type or vegetation structure [20]. Some species are more adapted to accessing aquatic oxygen and are therefore more capable at withstanding prolonged submersion by the tide [21]. An increasing tidal height could cause changes to all of these factors [22,23] and may result in a concertinaing of species.

The banana fiddler crab, *Austruca mjoebergi* (previously known as *Uca mjoebergi*), is one of the smaller species of fiddler crab (average carapace width = 11 mm, average claw length = 16 mm) located along the coast of Northern Australia and it inhabits the highest intertidal zone. In recent years, a larger species, *Tabuca elegans* (previously known as *Uca elegans*—average carapace width = 15 mm, average claw length = 23 mm), has begun to invade that area (P.R.Y.B. 2016, personal observation), providing a unique opportunity to study the effects of interspecific competition, as two previously spatially exclusive species are now co-occurring. While in other locations *A. mjoebergi* share a mudflat with other fiddler crab species [24], this particular population has always historically exclusively occupied the study area. This area has been studied since 2002 [25] and the history of the site is well documented ([26] and references cited within). Previous studies have looked at interactions between these two species after the initial arrival of *T. elegans* [27] but no study has attempted to assess the possible negative influences experienced by *A. mjoebergi* as a result.

When an individual of the larger invading species arrives in the population of *A. mjoebergi*, it will need to locate a burrow. We predict that *T. elegans* will evict *A. mjoebergi* upon settling within the *A. mjoebergi* population and all size classes of *A. mjoebergi* are at risk. Fight outcome is dependent on male size in most fiddler crab species including the study species, *A. mjoebergi* [17,19,25,28,29]. This suggests invading *T. elegans* may target smaller individuals (both smaller conspecifics and the smaller heterospecific *A. mjoebergi* males) to increase their chances of winning a territory. However, access to a burrow provides leverage during fights and retreat sites for residents resulting in a resident effect in fight outcome, which may aid residents in the defence of their territory [17,19].

Resource quality may also influence a *T. elegans* male's decision on who to fight for a new territory [7]. We know that resource quality is an important factor in conspecific fights [19,29]. If a male wins a fight with a smaller male, the burrow will need to be enlarged, adding to the cost of fighting a smaller rival [19,30], particularly if there is a large discrepancy between the size of the invading crab and the newly acquired burrow. This may be compounded if the burrows of different species have a different structure as is probably the case between *A. mjoebergi* and *T. elegans* burrows (H.L.C. 2016, personal observation).

Once territories have been won and territorial boundaries between neighbouring residents have been established, there may be both benefits and costs for resident *A. mjoebergi* to having a *T. elegans* neighbour. Over time, aggression between neighbouring individuals should decrease [17,28,31]. One benefit of having a heterospecific *T. elegans* neighbour is that they do not compete in female attraction. Another benefit is that *T. elegans* has been observed assisting smaller, neighbouring *A. mjoebergi* in territorial defence against another *T. elegans* [27]. However, heterospecifics can also have detrimental impacts [32]. For example, resident *A. mjoebergi* may suffer a reduction in territory size because new neighbours renegotiate territory boundaries through aggressive interactions [17,25,28,33], with the winner usually obtaining a small amount of extra territory [25,34].

This study aims to assess whether the smaller *A. mjoebergi* will experience any detrimental impacts as a result of the invasion by *T. elegans* by addressing the following questions. (i) Are *A. mjoebergi* residents evicted by larger *T. elegans* invaders? (ii) Do *T. elegans* preferentially evict heterospecific *A. mjoebergi* over conspecific *T. elegans*? (iii) Do resident *A. mjoebergi* suffer greater costs when residing next to a new *T. elegans* neighbour when compared to a new *A. mjoebergi* neighbour? (iv) Are there any ongoing costs for resident *A. mjoebergi* residing next to an established *T. elegans* neighbour compared to an established *A. mjoebergi* neighbour?

## 2. Material and methods

Research was conducted at East Point Coastal Reserve, Darwin, Australia (12°24^′^31.89^″^ S and 130°49^′^49.12^″^ E) between the months of September and December of both 2013 and 2014. As we were assessing impacts on *A. mjoebergi*, all data were collected at all times of the day during the neap tides, which is the peak mating phase for *A. mjoebergi* [26,35], while *T. elegans* mate during spring tides [36]. All carapace and claw measurements were made with the use of callipers to the nearest 0.1 mm. If a crab had to be held out of the burrow for any length of time, it was placed in a small cup of water in a shaded area to prevent it from overheating.

### 2.1. Burrow acquisition

A total of 40 arenas were constructed by surrounding naturally occurring individuals with temporary plastic collars (20 cm high and 40 cm diameter). Half of the arenas were set up around three naturally occurring male *A. mjoebergi* individuals that were specifically chosen for their relative sizes: one was large, one medium and one small. The remaining half of the arenas were placed around two naturally occurring males, one *A. mjoebergi* and one *T. elegans* male. In each of the 40 arenas, we individually added a single *T. elegans* male by capturing him in an area of the mudflat away from the experimental arenas, measuring him and placing him in an upturned clear plastic container within the arena. When all the natural arena occupants were surface-active (approx. 2 min), the ‘intruding’ *T. elegans* was remotely released by pulling a cable connected to the release container. After 1 h, it was noted which individual (if any) the invader had evicted from their burrow. Burrow sizes of the naturally occurring residents were noted as burrow entrance diameter is closely correlated with resident size [37].

### 2.2. Neighbour interactions: new neighbours

We selected 25 naturally occurring pairs of *A. mjoebergi* male neighbours (abutting territories), and captured one of the pair. In 13 trials we replaced the neighbour with a *T. elegans* male, and in 12 trials we replaced him with an *A. mjoebergi* male. We video-recorded each pair for 30 min after they both re-emerged from their burrows and were surface-active. Trials ended early if either the resident or intruding neighbour left voluntarily, were evicted, or sealed themselves into their burrow. From the videos, we counted the number of fights as well as the fight escalation level: display, push or grapple. The ‘display’ phase is the first stage and represents the lowest escalation level: males face each other and wave. The ‘push’ is the second stage and is medium escalation: males align their claws face-on and push each other. The final stage, high escalation, is grappling: males interlock their claws and grapple with each other. Each fight was assigned a score based on the maximum escalation level of the fight (display = 1; push = 2 and grapple = 3). The total duration of each fight was recorded as well as durations of each escalation level within each fight. Claw and carapace sizes were recorded for each of the crabs. Distance between the two burrows was also noted.

### 2.3. Neighbour interactions: established neighbours

Naturally occurring, established male neighbours were assessed by observing 44 naturally occurring pairs of crabs; half of them were pairs of *A. mjoebergi* neighbouring males and half were pairs in which one male was an *A. mjoebergi* and the other was a *T. elegans* male. In the pairs of *A. mjoebergi* males, the smaller of the males was assigned to be the focal male and the other to be the neighbour (as *T. elegans* is larger than *A. mjoebergi*, this meant that the focal male was always the smallest male in conspecific and heterospecific trials). For each male in each pair, we documented the total amount of time the male spent surface-active and the amount of time spent in the burrow over a 30 min observation period. We measured the size of each crab and the distance between their burrows.

#### 2.3.1. Statistical analysis

All statistics were conducted using IBM SPSS 22. We used carapace width as a measure of size as carapace width and claw length are highly correlated [37]. Arena data were assessed with correlations. Neighbour interaction data were assessed with Mann–Whitney *U*-non-parametric tests as the data distribution could not be normalized. Where multiple correlations were run on the same data, the raw *p*-values were used in a Benjimini–Hochberg false discovery rate (FDR) test with an FDR of 15%. Raw *p*-values are presented with their significance against the FDR-adjusted *α*-levels brackets.

Owing to unpredictable crab behaviour, trial lengths varied during the neighbour interaction trials and the data were weighted to account for these differences. Some trials were too short to be included (under 5 min) and thus were removed from the analysis. Information unaffected by trial length (i.e. crab size and distance) was left in the analysis as this information was used in supporting analysis such as correlations. As a result, there are differing *N* values within the same set of analysis on some tests.

## 3. Results

### 3.1. Burrow acquisition

Resident male *A. mjoebergi* were evicted by intruding male *T. elegans*. In the 20 experiments where a *T. elegans* male was placed in an arena with three naturally occurring *A. mjoebergi* males (one large, one medium and one small); the intruding *T. elegans* male evicted one of the residents in 15 cases (75%). In eight cases the large *A. mjoebergi* male was evicted, in one case the medium male was evicted, and in six cases it was the smallest *A. mjoebergi* male that was evicted. There was no correlation between the size of the introduced *T. elegans* male and the burrow diameter of the evicted *A. mjoebergi* male (Pearson's *r* = 0.186, *p* = 0.508, *n* = 15).

The rate at which *A. mjoebergi* individuals are evicted by intruding *T. elegans* is halved when residing next to a heterospecific male as large *T. elegans* are equally likely to evict a conspecific as a heterospecific. In the 20 experiments where a *T. elegans* male was introduced into an arena with a naturally occurring pair of males, one *A. mjoebergi* and one *T. elegans*, there were 13 evictions: in five cases the introduced *T. elegans* male evicted an *A. mjoebergi* male, and in eight cases he evicted a *T. elegans* male. This is a non-significant difference (binomial *p* = 0.58; *n* = 5, 8). There was no correlation between invader size and the size of the evicted individual's burrow (Pearson's *r* = −0.521, *p* = 0.068, *n* = 13). Naturally occurring *T. elegans* were significantly larger than their *A. mjoebergi* neighbours (Mann–Whitney *U*-test: *Z* = −2.963, *p* = 0.003, *n* = 20, range = 8.1–14.2, mean = 10.95, s.d. = 1.82).

### 3.2. Neighbour interactions: new neighbours

When residing next to a new heterospecific neighbour, *A. mjoebergi* are at a greater disadvantage compared with those residing next to a new conspecific neighbour. When a resident *A. mjoebergi* male had his naturally occurring conspecific neighbour replaced by a *T. elegans* male, they engaged in significantly more fights (Mann–Whitney *U* test: *Z* = −2.268, *p* = 0.022, *n* = 12; 13, range = 0–37, mean = 9.35, s.d. = 11.25) and spent more time in low escalation level (display) fights (Mann–Whitney *U*-test: *Z* = −2.184, *p* = 0.03, *n* = 12; 13, range = 0–502, mean = 85.75, s.d. = 123.18) than when his neighbour was replaced with another *A. mjoebergi* male. There was no significant difference between the total fight time (Mann–Whitney *U*-test: *Z* = −1.775, *p* = 0.077, *n* = 12; 13, range = 0–554, mean = 126.38, s.d. = 148.98), number of fights reaching any of the escalation levels (Mann–Whitney *U*-test: *Z* = −1.994, *p* = 0.06, *n* = 12; 13, range = 0–3, mean = 1.84, s.d. = 1.18), total time spent push fighting (Mann–Whitney *U*-test: *Z* = −1.671, *p* = 0.11, *n* = 12; 13, range = 0–144, mean = 30.19, s.d. = 43.6), or total time spent grapple fighting (Mann–Whitney *U*-test: *Z* = −1.321, *p* = 0.247, *n* = 12; 13, range = 0–43, mean = 7.42, s.d. = 12.59) when the naturally occurring neighbour was replaced with a conspecific or a heterospecific male.

**Figure 1.**
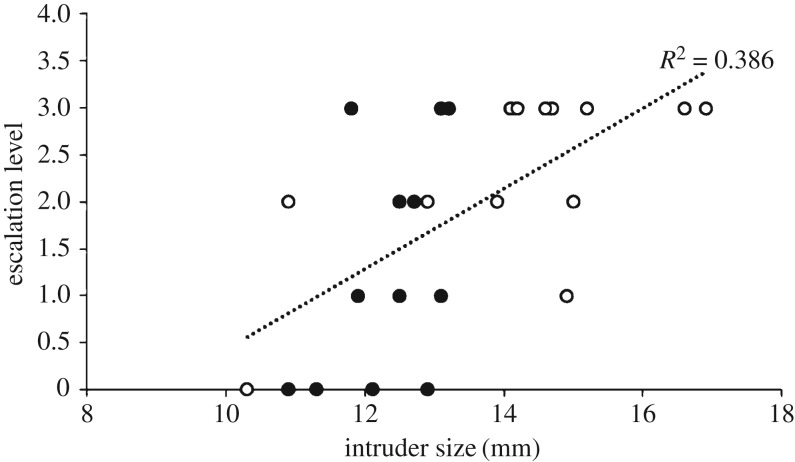
Fight escalation level when compared to intruding neighbour size (black dots, *A. mjoebergi* intruding neighbour; white dots, *T. elegans* intruding neighbour).

*Tabuca elegans* males are usually larger than *A. mjoeberg*i males, and as the relative size of rivals affects fight escalation, we found a positive correlation between the size of the new neighbour and the escalation level of fights (Pearson's *r* = 0.621, *p* = 0.001, *n* = 25, significant using the FDR-adjusted *α*-value of 0.006; figure 1). As focal size increases, so too does the time spent push fighting (Pearson's *r* = 0.506, *p* = 0.010, *n* = 25, significant using the FDR-adjusted *α*-value of 0.0125). There was no correlation found between any of the other tested variables (all *p* > 0.031, all non-significant using the FDR-adjusted *α*-values).

### 3.3. Neighbour interactions: established neighbours

There are some disadvantages faced by *A. mjoebergi* residents residing next to established heterospecific neighbours compared to *A. mjoebergi* individuals residing next to established conspecific neighbours. When examining naturally occurring neighbouring males (i.e. not interfered with, nor relocated by us, therefore we had no influence on the sizes of neighbouring individuals), we found that the established neighbour was larger when it was a *T. elegans* male than when it was an *A. mjoebergi* male (Mann–Whitney *U*-test: *Z* = −5.401, *p* = 0.000, *n* = 22; 22, range = 8.5–17.6, mean = 12.9, s.d. = 2.12). The distance between the burrows of neighbours was significantly larger when the neighbour was a heterospecific (rather than a conspecific) male (Mann–Whitney *U*-test: *Z* = −2.136, *p* = 0.033, *n* = 22; 22, range = 27.2–152.8, mean = 76.39, s.d. = 32.48) suggesting a loss of territory to *A. mjoebergi* residents. Established *T. elegans* neighbours spent significantly more time in their burrows than did the established (i.e. not the focal) *A. mjoebergi* neighbours (Mann–Whitney *U*-test: *Z* = −2.569, *p* = 0.01, *n* = 21; 21, range = 0–1449, mean = 330.96, s.d. = 326.21). There was no significant difference between the time the focal males spent in their burrows (Mann–Whitney *U*-test: *Z* = −0.239, *p* = 0.811, *n* = 20, range = 0–1537, mean = 407.53, s.d. = 325.55), or the total time spent interacting (Mann–Whitney *U*-test: *Z* = −0.383, *p* = 0.701, *n* = 12, range = 0–1329, mean = 177.83, s.d. = 297.69) when they had heterospecific or conspecific neighbours.

**Figure 2.**
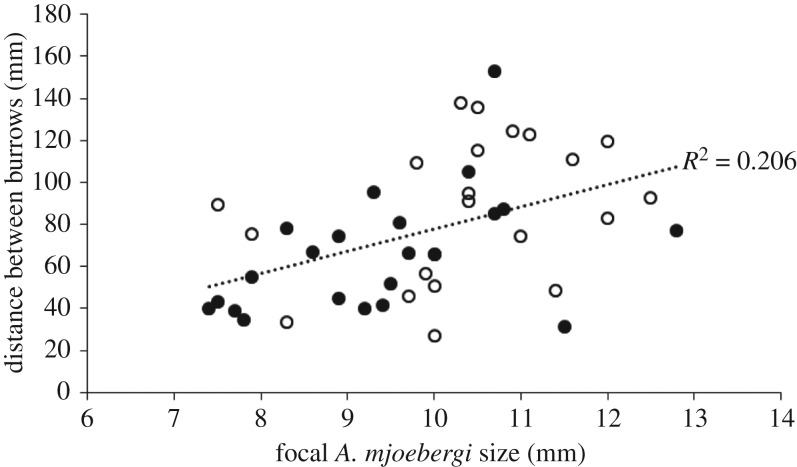
Distance between burrows of resident *A. mjoebergi* and established neighbours when compared to the size of the small resident *A. mjoebergi* (black dots, *A. mjoebergi* living next to an established *A. mjoebergi* conspecific; white dots, *A. mjoebergi* living next to an established *T. elegans* heterospecific).

**Figure 3.**
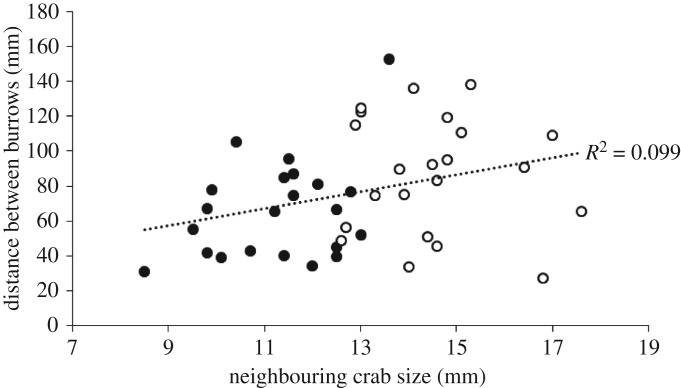
Distance between burrows of resident *A. mjoebergi* and established neighbours when compared to the size of the established neighbour (black dots, *A. mjoebergi* established neighbour; white dots, *T. elegans* established neighbour).

**Figure 4.**
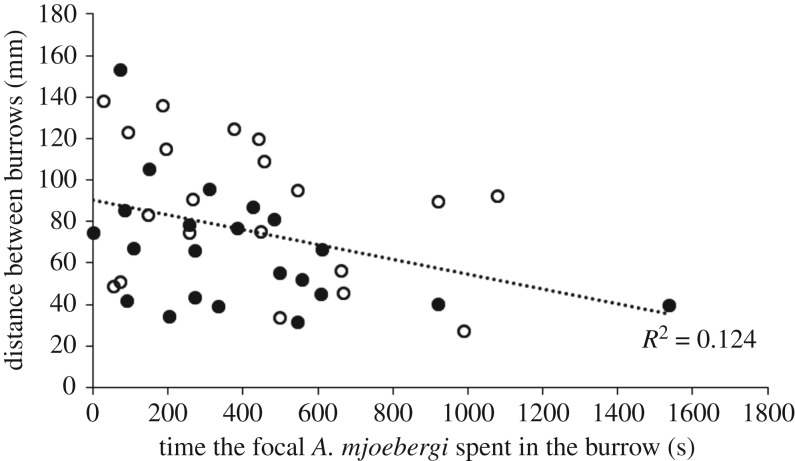
Effects of the distance between resident *A. mjoebergi* burrow and established neighbour burrow on the time the smaller resident *A. mjoebergi* spent in the burrow (black dots, *A. mjoebergi* established neighbour; white dots, *T. elegans* established neighbour).

As the size of the focal *A. mjoebergi* males increased, so too did the distance between the burrows (Pearson's *r* = 0.454, *p* = 0.002, *n* = 44, significant using the FDR-adjusted *α*-value of 0.025; figure 2). Similarly, as the size of the established neighbour increased, so too did the distance between the burrows (Pearson's *r* = 0.315, *p* = 0.037, *n* = 44, significant using the FDR-adjusted *α*-value of 0.075; figure 3). As the distance between the burrows increased, the smaller resident spent less time in the burrow (Pearson's *r* = −0.353, *p* = 0.022, *n* = 42, significant using the FDR-adjusted *α*-value of 0.05; figure 4). No other correlations were observed between the other tested variables (all *p* > 0.26, all non-significant using the FDR-adjusted *α*-values).

## 4. Discussion

Fiddler crab distribution can be quite complex. In some locations, species seldom overlap in distribution as each species is adapted to very particular sediments, micro-climates and intertidal zones [20,21]. In other locations, many species can coexist at one time including the focal species of this study, *A. mjoebergi* [24]. Our study population of *A. mjoebergi*, however, has historically existed as a single species population but recently (over the past 10 years) has increasingly been ‘invaded’ by a larger fiddler crab species, *T. elegans* (P.R.Y.B. 2016, personal observation).The overlap we currently observe therefore provides an ideal opportunity to study interspecific interactions, especially since *T. elegans* are much larger than *A. mjoebergi* and are likely to out-compete them.

Invading *T. elegans* will fight and in up to 75% of cases successfully evict *A. mjoebergi* for access to territories. Fiddler crabs usually target smaller individuals in territorial fights in order to improve their chances of winning the fight [17,19,25,28,29]. However, invading individuals may also target individuals of a similar size to themselves, to avoid excessive burrow modifications after a territory has been won [19,30]. Neither of these factors appears to play a role when invading *T. elegans* evict *A. mjoebergi*, as all size classes were evicted.

When given a choice of fighting with a conspecific or a heterospecific male, *T. elegans* are non-selective: they are just as likely to attack an *A. mjoebergi* as a *T. elegans* resident. This is interesting since the two species potentially have different burrow structures (H.L.C. 2016, personal observation) which would represent different resource qualities and should, therefore, affect the male's decision on who to fight [7]. However, *T. elegans* evicted both conspecifics and heterospecifics. While *A. mjoebergi* were not specifically targeted by *T. elegans* intruders, they were also not avoided.

Once an invader has acquired a burrow, negotiations with the surrounding new neighbours over territorial boundary lines begin [17,25,28,33]. Here, we show that *A. mjoebergi* residents engage in a greater number of low escalation aggressive displays when they gain a new, heterospecific territorial neighbour than when their new neighbour is a conspecific male. This is similar to results found by Aspey [38], who found *U. pugnax* had greater levels of antagonistic displays towards *U. pugilator* neighbours than *U. pugilator* showed towards *U. pugnax*.

After territorial boundaries have been set, aggression between neighbours dissipates [17,28,31]. Here, we found that there was a greater distance between burrows of established heterospecific neighbours compared with established conspecific neighbours (two *A. mjoebergi*), and this distance was related to the sizes of both of the neighbouring individuals (figures 2 and 3). This indicates that *T. elegans* occupy larger territories than *A. mjoebergi* either due to their larger size (hence their increased requirement for feeding space) or due to their increased aggression, reducing the available space for the original, resident species. This has been found in other fiddler crab species; for example, the number of burrows dug by *U. pugilator* and *U. minax* were reduced by 50% and 20%, respectively, when residing in mixed species populations. These reductions were attributed to competition with other species among other factors [20].Territory sizes and resultant impacts for both *A. mjoebergi* and *T. elegans* need further investigation and this is the focus of another study.

Established *T. elegans* neighbours spent more time in the burrow than the established *A. mjoebergi* neighbours. It would seem that this would translate into more available time on the surface, feeding and courting, for those individuals residing next to an established *T. elegans* neighbour; however, there was no difference in time spent down between resident *A. mjoebergi* living next to established heterospecifics when compared to individuals living next to established conspecifics. This suggests that, once territorial boundaries have been set, *A. mjoebergi* males do not appear to be at a disadvantage when having a conspecific or a heterospecific neighbour. Resident *A. mjoebergi* also spent more time in the burrow, however, the closer the two burrows were to each other (figure 4).

## 5. Conclusion

Common sense suggests that individuals of a smaller species will be disadvantaged when their population is invaded by a larger species that uses the same resources. Here, we show that common sense can be wrong. There was very little effect on the small *A. mjoebergi* males when they live among conspecifics and when their neighbours are the larger, heterospecific *T. elegans* males. While this study is a small snapshot in time, we believe the assertion that the effects on *A. mjoebergi* are minimal to be accurate, since many fiddler crab species regularly coexist in mixed species populations [24,38] including *A. mjoebergi* [24]. In fact, once territorial boundaries have been established, studies have revealed that *A. mjoebergi* may benefit from having a heterospecific neighbour (i.e. reduced competition for mates and assistance in heterospecific fights [29]). This is an interesting point since niche theory suggests that in order to coexist, two species must diverge in their use of resources [39]. By developing coalitions that promote the existence of mixed species populations, it is possible that the predicted outcomes of traditional niche and competition theories have been weakened.

This study has demonstrated that interspecific competition need not have the negative impacts on individuals as shown by Guiasu & Dunham [10] and may not necessarily result in local extinction of species. This is true even when the species live in high-density, mixed species populations that are forced to compete for limited resources that are required for survival [4,5,40]. When two sympatric species occupying the same or similar niches and are forced to interact, one often emerges as the dominant species at the expense of the other [8–10], but not always. In a world with a changing climate that will result in a reduction of available habitat for many species [2], it is imperative we understand the consequences of species overlap so as to understand the true impact on biodiversity and species survival.

## Data accessibility

Our data are deposited at the Dryad Digital Repository: http://dx.doi.org/10.5061/dryad.k3c3v [41].
